# Enteroparasites in Riverside Settlements in the Pantanal Wetlands Ecosystem

**DOI:** 10.1155/2018/6839745

**Published:** 2018-01-16

**Authors:** Patrícia Vieira da Silva, Lucimare dos Santos Maciel, Ludiele Souza Castro, Paula Guerra Murat, Minoru German Higa Junior, Patrícia Honorato Zerlotti, Ana Rita Coimbra Motta-Castro, Elenir Rose Jardim Cury Pontes, Maria Elizabeth Cavalheiros Dorval

**Affiliations:** ^1^Postgraduate Program in Infectious and Parasitic Diseases, Universidade Federal de Mato Grosso do Sul, Campo Grande, MS, Brazil; ^2^Ecologia e Ação (ECOA), Campo Grande, MS, Brazil

## Abstract

**Background:**

Intestinal parasites are a major source of health problems in developing countries, where socioeconomic, cultural, and environmental conditions contribute in maintaining the biological cycles of various parasites and facilitating their spread. The objective of this study, conducted in Corumbá, Mato Grosso do Sul state, Brazil, was to investigate the occurrence of intestinal parasites in riverside communities in the South Pantanal wetlands and conduct educational interventions focused on health and environmental preservation.

**Method:**

In total, 196 stool samples were tested for parasites using the merthiolate-iodine-formaldehyde concentration (MIFC) technique and spontaneous sedimentation and educational activities were carried out.

**Results:**

Enteroparasite prevalence was 72% (65.6–78.2%; 95% CI). Of the 141 positive cases, monoparasitism was found in 34.7%, biparasitism in 23%, and polyparasitism in 14.3%.* Entamoeba coli* was the most frequent protozoan (70.2%). Among helminths, hookworms were the most prevalent. Enteroparasitosis prevalence did not differ for sex or place of abode but proved higher in individuals older than 10 years.

**Conclusion:**

The high positivity rate for enteroparasites found for the communities stems from lack of sanitation and poor personal and environmental hygiene habits, indicating that effective health policies and educational interventions are needed to reduce the current risk levels.

## 1. Introduction

Intestinal parasites are a major debilitating factor, often associated with chronic diarrhea and malnutrition, that can compromise both physical and intellectual development, particularly in younger age groups [1, 2].

Intestinal parasites are a major source of health problems in developing countries, where socioeconomic, cultural, and environmental conditions contribute in maintaining the biological cycles of parasites and facilitating their spread [3, 4].

Furthermore, the escalation of predatory practices against the environment, compounded by detrimental aspects of political and economic frameworks, collaborates to increase the occurrence of parasitic and other diseases [5, 6].

Despite the current status of the Pantanal wetlands as a World Natural Heritage site, owing to its vast biogeographical area and great biodiversity of plant and animal species, many human residents in the region experience difficulties that hamper survival, including the absence of sanitation and healthcare services.

The aim of this study was to investigate the occurrence of enteroparasites among dwellers of three riverside communities in the Pantanal ecosystem, seeking to elucidate key epidemiological factors involved in transmission and perform educational interventions to raise awareness on enteroparasitosis prevention and environmental preservation.

## 2. Materials and Methods

**Figure 1:**
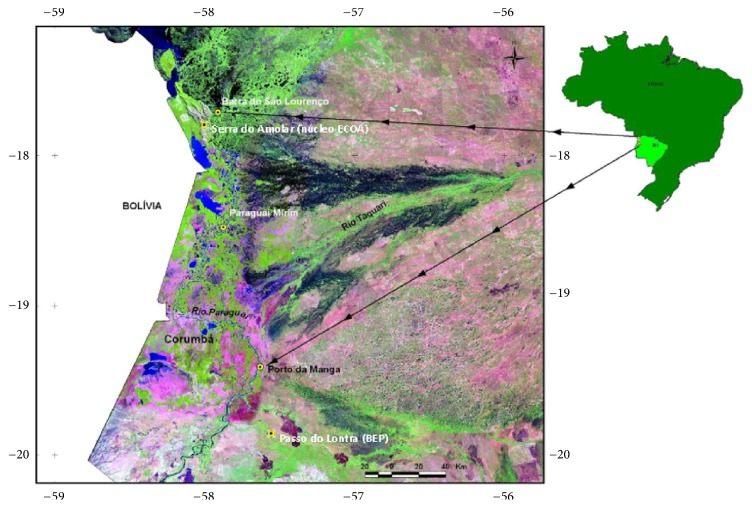
Location of the communities investigated. Source: ECOA, 2010.

For this analytical cross-sectional study based on primary data and benchwork research, parasitological stool tests were performed among dwellers of three riverside settlements from July 2008 to July 2009. The communities studied—Barra de São Lourenço, Paraguai Mirim, and Porto da Manga—are located on the Paraguai river in Corumbá county, Mato Grosso do Sul state. Barra de São Lourenço (17°54′0′′S; 57°27′39′′W) and Paraguai Mirim (21k 0454391; UTM 7953009) can only be reached via a 250 km boat trip from Corumbá, while Porto da Manga (19°15′33.15′′S; 57°14′7.13′′W), 385 km away from Campo Grande (the state capital), is served by the Parque Pantanal state highway (Figure 1).

This study was part of the project entitled* Crianças das Águas, Pantanal: Identidade e Cidadania [Children of the Waters, Pantanal: Identity and Empowerment]*, supported by the Criança Esperança Program (a privately sponsored social mobilization initiative in partnership with the UNESCO). All residents of the area were invited; a total of 196 adults and children (male and female) who voluntarily agreed to participate and provided written informed consent were enrolled in the study. Sample size (*n* = 196) was calculated in the Epi-Info version 7 program, according to the following parameters: population of 400 residents, prevalence of 50% (±5%), and significance level of 5%.

The project was approved by the Universidade Federal de Mato Grosso do Sul (UFMS) Committee for Ethics in Research in Humans (permit 1612). For the participation of minors (under 18), written approval was obtained from parents or guardians.

Stool samples were collected in merthiolate-iodine-formaldehyde solution, stored in suitable containers, and tested at the UFMS Laboratory of Clinical Parasitology using techniques proposed by Blagg et al. [7] (merthiolate-iodine-formaldehyde concentration) and Hoffmann et al. [8] (spontaneous sedimentation). Individuals diagnosed with pathogenic parasites were prescribed specific treatment. The educational activities consisted of lectures, a stage play presentation, and individual guidance.

The data were initially treated using descriptive statistics. Chi-squared and chi-squared trend tests were employed to detect associations between variables at a significance level of 5%. The data were keyed into in Microsoft Excel 2010 spreadsheets (Microsoft, Redmond, WA, USA) and treated using Epi-Info 7.1.1.14 (Centers for Diseases Control and Prevention, Atlanta, GA, USA) and BioEstat 5.3 (Sociedade Mamirauá, Belém, PA, Brazil) software.

## 3. Results

The study comprised 196 subjects (83 male, 42.3%; 113 female, 57.7%). Age ranged from 10 months to 88 years.

Participants reported lack of basic sanitation and garbage collection in the communities (Paraguai Mirim, *n* = 110; Barra de São Lourenço, *n* = 50; Porto da Manga, *n* = 36). The Paraguai river was their primary water source. The settlements are extremely poor: household income is typically below one Brazilian minimum wage. All garbage is dumped into the river or discarded in the open. Some families use sodium hypochlorite to disinfect water. Houses, made of wood or wattle and daub, are covered with thatch or corrugated fiber cement sheets. Floors are not tiled. There are no bathrooms. Adults work as bait sellers, fishermen, and boat skippers.

No difference was observed between males and females in the prevalence of enteroparasite infection (*p* = 0.383). Prevalence was higher, however, in individuals older than 10 years (*p* = 0.001) (Table 1).

No significant association (*p* = 0.930) was found between infection prevalence and place of abode (Table 1).

**Figure 2:**
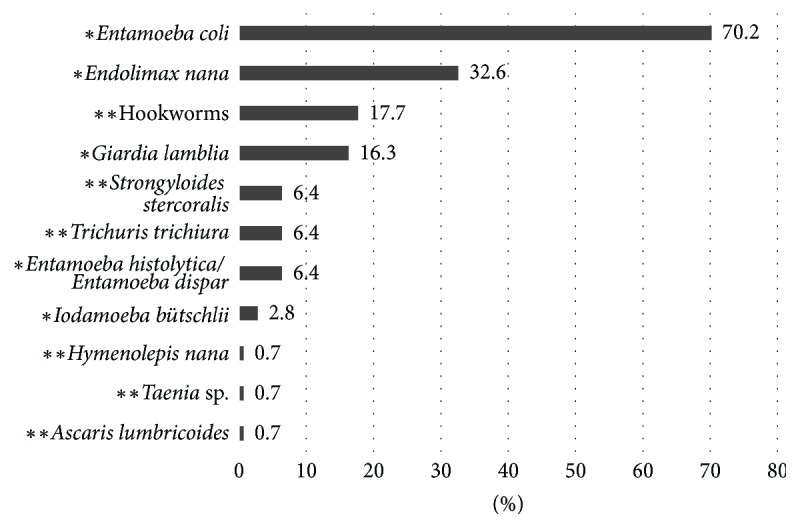
Distribution of subjects (*n* = 141), by enteroparasite species detected (^*∗*^protozoans; ^*∗∗*^helminths).

Of the samples investigated, 72% tested positive for enteroparasites (65.6–78.2%, 95% CI). Eleven species were identified, comprising 79.7% protozoa* (Giardia lamblia*,* Entamoeba histolytica/Entamoeba dispar*,* Entamoeba coli*,* Endolimax nana*,* Iodamoeba bütschlii)* and 20.3% helminths* Hymenolepis nana*,* Strongyloides stercoralis*,* Trichuris trichiura*,* Ascaris lumbricoides, *hookworms, and* Taenia* sp. (Figure 2).

Among the 141 individuals infected, monoparasitism (48.2%) prevailed over cases of biparasitism (31.9%) or polyparasitism (19.9%). Infection by protozoa predominated.

*Entamoeba coli* was the most prevalent parasite (70.2% of the 141 positive cases), followed by* Endolimax nana *(32.6%), hookworms (17.7%),* Giardia lamblia *(16.3%),* Strongyloides stercoralis *(6.4%),* Trichuris trichiura *(6.4%),* Entamoeba histolytica/Entamoeba dispar *(6.4%),* Iodamoeba bütschlii *(2.8%),* Hymenolepis nana* (0.7%),* Taenia* sp. (0.7%), and* Ascaris lumbricoides *(0.7%) (Figure 1).

Seven pathogenic organisms (hookworms,* Giardia lamblia, Trichuris trichiura, Strongyloides stercoralis, Ascaris lumbricoides, Taenia* sp.,* Hymenolepis nana*), three commensal species* (Endolimax nana, Iodamoeba bütschlii, Entamoeba coli)* and one undefined (*Entamoeba histolytica/Entamoeba dispar* complex) were detected.

## 4. Discussion

Distance from urban centers and poor transportation isolate these communities from mainstream society and hamper their access to healthcare services. Unfavorable socioeconomic conditions, lack of sanitation, and educational shortcomings may explain the high prevalence of enteroparasites [4, 9]. Economically disadvantaged social groups are more prone to a number of diseases, including enteroparasitosis associated with unsanitary conditions that contaminate waterbodies, facilitating the accumulation of waste, including fecal matter from diseased residents and asymptomatic carriers. These environmental conditions promote the proliferation of insects and rodents that act as mechanical vectors for parasites [10, 11].

A high prevalence of enteroparasites was also reported in 1994 by Coura et al. [12] for riverside settlements in Amazonas state, with 75.5% positive cases among 441 tests performed at a 95% confidence level. More recently, investigating a riverside community in Coari county, Amazonas state, Santos et al. [5] detected 83% positive cases among children.

Investigating 91 samples from riverside residents in Igarapé Miri county, Pará state, Silva et al. [13] found a 94.5% positivity rate, with a higher prevalence of enteroparasites in children and adolescents (71.4%), and a higher frequency of* Ascaris lumbricoides* (57.14%) and* Trichuris trichiura* (41.76%) helminths. In a previous study [14] of samples from 65 children aged 1–12 years living in the riverside community of San Francisco do Laranjal, in Coari county, an 83.1% rate of enteroparasite infection was detected.

In the present study, protozoans predominated (79.7%), particularly* Entamoeba coli* (70.2%), a commensal parasite. Its high prevalence is indicative of poor sanitary and socioeconomic conditions, since its fecal-oral transmission also increases the risk of contamination by other pathogens transmitted through the same route [15]. Consumption of untreated water makes riverside settlements more susceptible to waterborne diseases, a feature aggravated by the absence of water supplies other than rivers, into which sewage and other waste flows. These conditions perpetuate the transmission cycles of enteroparasites and other waterborne infective agents [16].

Among helminths, the predominance of hookworms raises concern, given their hematophagous behavior. Particularly in children, infection can lead to iron-deficiency anemia and hypoproteinemia, with subsequent edema, atrophy of the intestinal mucosa, and decreased absorption [3, 17]. A high prevalence of hookworms has been reported for other riverside populations. Proximity to water, combined with a humid, warm climate, sandy soil, and poor sanitation, creates favorable conditions for larval development and persistence in the environment [18, 19].

Despite a predominance of monoparasitism, cases of polyparasitic infection require attention, given their high probability of including pathogenic species.

No significant differences in infection prevalence were found for place of abode or sex; this result was confirmed by some studies [20]. The high positivity rate for enteroparasites, whether pathogenic or commensal reflects the exposure of these communities to soil and water contamination and their poor hygiene habits, demonstrating that transmission and maintenance of parasitic infections are part of an interactive process involving infective agent, environment, and susceptible host.

## 5. Conclusion

The high positivity rate for enteroparasites detected in these riverside settlements in the South Pantanal wetlands reflects the absence of basic sanitation and poor personal and environmental hygiene habits.

This study, the first to investigate the occurrence of enteroparasitosis in riverside communities in this region, revealed the need for the implementation of effective measures in environmental and health education, as well as investment in sanitation infrastructure, to improve the quality of life of this population.

## Figures and Tables

**Table 1:** Distribution of subjects, by presence or absence of enteroparasites, sex, age, and place of abode (*n* = 196).

Variable	Enteroparasites				*p*	PR (95% CI)
	Present		Absent			
	*N*	%	*N*	%		
Sex						
Female	84	74.3	29	25.7	^(1)^0.383	1
Male	57	68.7	26	31.3		1.08 (0.90–1.30)
Age range (years)						
>40	16	94.1	1	5.9	^(2)^ **0.001**	1
21–40	22	78.6	6	21.4		1.20 (0.95–1.50)
11–20	23	79.3	6	20.7		1.19 (0.95–1.48)
6–10	34	72.3	13	27.7		1.30 (1.05–1.61)
≤5	16	50.0	16	50.0		1.88 (1.31–2.72)
No data	30	69.8	13	30.2		-
Place of abode						
Paraguai Mirim	80	72.7	30	27.3	^(1)^0.930	1
Barra de São Lourenço	36	72.0	14	28.0		1.01 (0.82–1.24)
Porto da Manga	25	69.4	11	30.6		1.05 (0.82–1.34)

PR: prevalence ratio; ^(1)^chi-squared test; ^(2)^chi-square test of trends. Category “no data” was excluded from statistical calculations.
